# 1562. Renal Outcomes after Switch from Oral Antiretroviral therapy (ART) to Long-acting injectable Cabotegravir/Rilpivirine (LAI CAB)

**DOI:** 10.1093/ofid/ofad500.1397

**Published:** 2023-11-27

**Authors:** Alvaro Ayala, Lukas McNaboe, Julia Kostka, Lisa M Chirch

**Affiliations:** University of Connecticut, Farmington, Connecticut; University of Connecticut, Farmington, Connecticut; University of Connecticut, Farmington, Connecticut; University of Connecticut School of Medicine, Farmington, CT

## Abstract

**Background:**

In previous studies, patients switching from Tenofovir Disoproxil (TDF) to Long Acting Injectable (LAI) Cabotegravir/Rilpivirine demonstrated improvement in renal outcomes. Furthermore, Tenofovir Alafenamide (TAF) has shown improved renal outcomes compared TDF. In this study, we aim to compare Estimated Glomerular Filtration Rate (eGFR) changes in patients who switched from Integrase Inhibitors (INSTI), with and without a TAF based regimen oral therapy, to LAI.

**Methods:**

Demographics, previous antiretroviral therapy regimen, and clinical parameters were collected at the initiation of LAI (T0), 2 months (T1), 6 months (T2), and 12 months before and after the switch to LAI. Patients who remained on TAF-containing regimens only were compared to patients who switched to LAI after they were previously on TAF. eGFR using CKD epi formula was utilized in order to compare these groups. A multiple linear regression adjusted for variables impacting renal outcomes was performed for all patients, and patients previously on TAF.

**Results:**

A total of 22 patients on LAI and 44 on oral INSTI-based regimens were included. There was a difference in age for those who switched to LAI compared to those who remained on oral therapy (Table 1). Demographic data for patients previously on TAF is shown in Table 2. There was no significant difference between eGFR in all patients and the TAF subgroup who switched to LAI at 2 months, 6 months, and 12 months respectively, (Figure 1 & 2). When adjusting for other variables, there was no significant change in eGFR between LAI vs oral in both overall and TAF groups at 12 months, (Tables 3 & 4).

**Figure:**
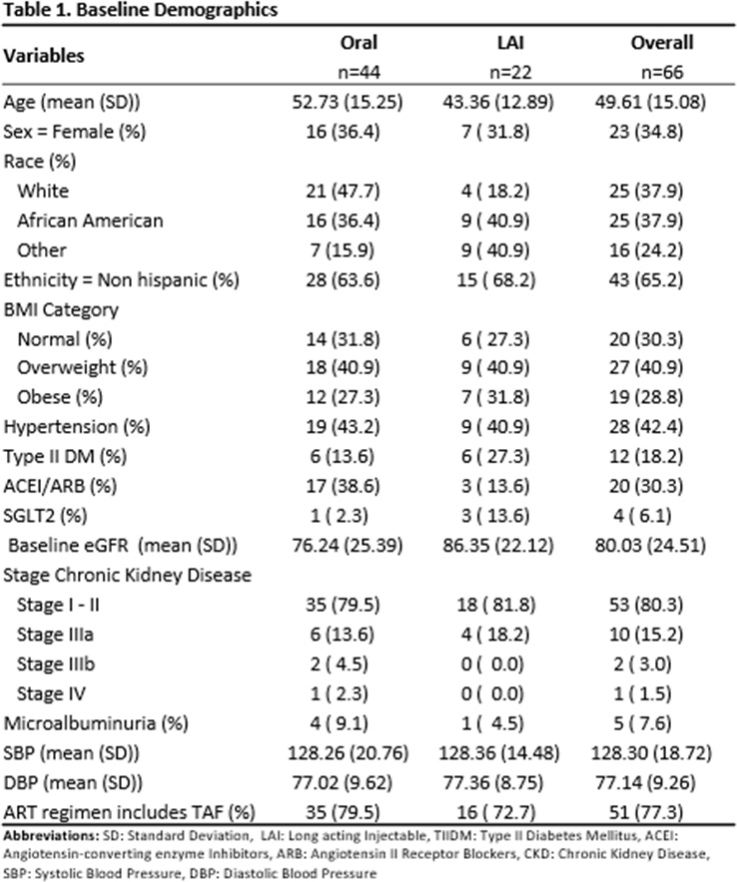

**Figure:**
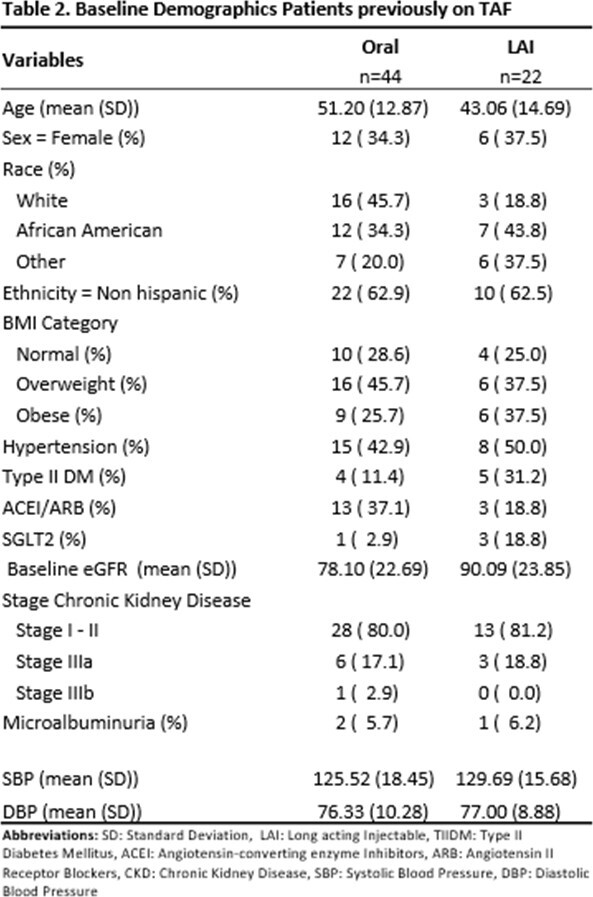

**Figure:**
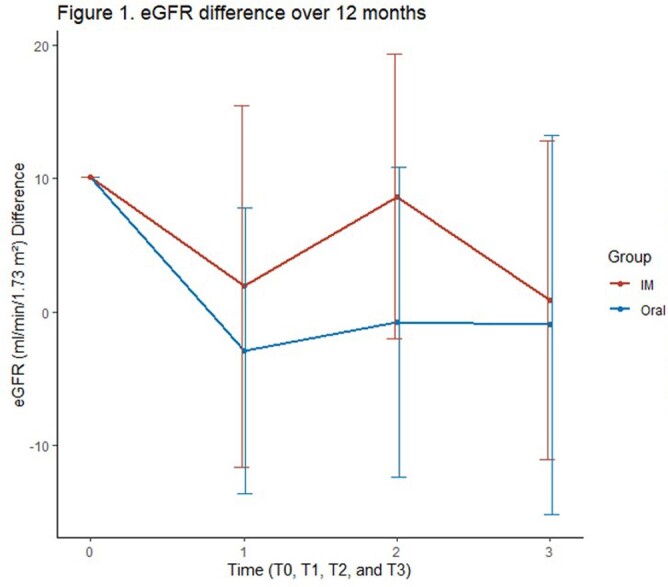

**Figure:**
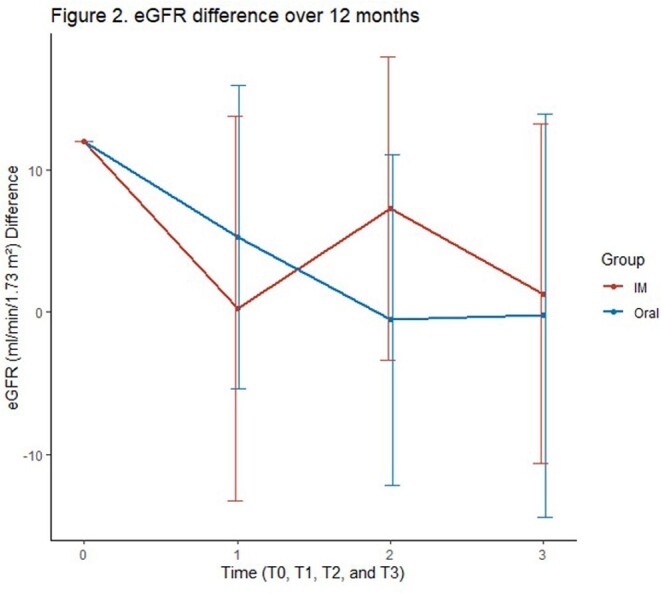

**Figure:**
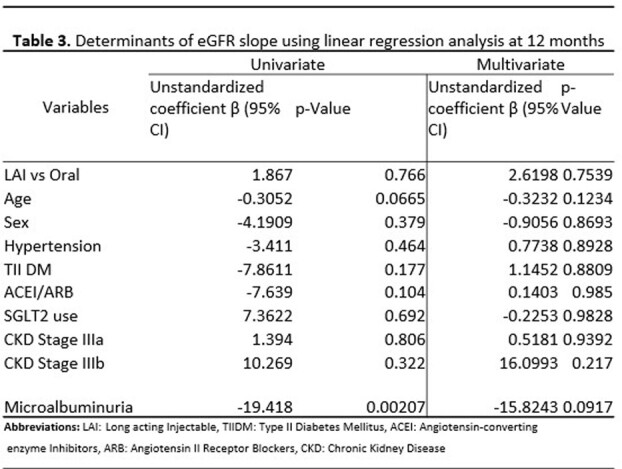

**Figure:**
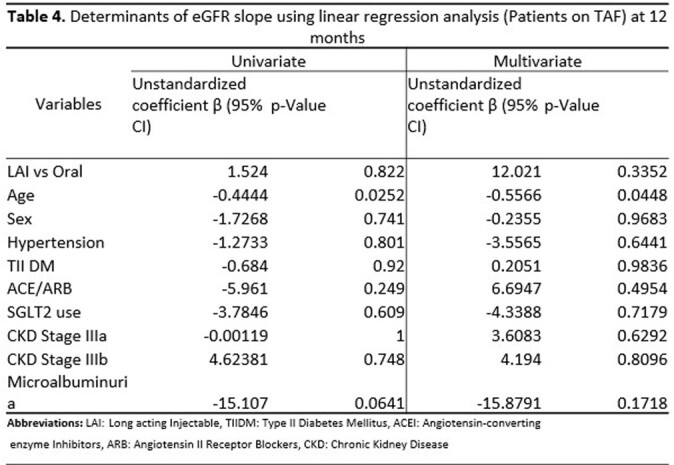

**Conclusion:**

In this retrospective study, there was no significant change in eGFR between patients starting on LAI and those who continued on an oral regimen with and without TAF. Further real-world data assessment for long-term renal outcomes in patients on LAI is needed.

**Disclosures:**

**All Authors**: No reported disclosures

